# A bi-objective game-theoretic model for collaboration formation between software development firms

**DOI:** 10.1371/journal.pone.0219216

**Published:** 2019-07-10

**Authors:** Muhammad Fahimullah, Yasir Faheem, Naveed Ahmad

**Affiliations:** Department of Computer Science, COMSATS University Islamabad, Islamabad Campus, Pakistan; Shandong University of Science and Technology, CHINA

## Abstract

Requirement for formation of collaborations has been on increase for the software development industry, especially for smaller to medium sized firms, due to rapid technological advancements, requirements for diversified skills, ever enhancing demands for innovation and fierce competition. Collaborative product development in an alliance enables the firms to benefit from each other’s diversified skills and the experience as a result of which they can develop products more rapidly and of better quality as well resulting in a higher payoff. Also, the development costs decrease. However, to avoid undesired results, selection of an appropriate partner firm for collaboration is of utmost importance keeping in view the objectives of alliance formation of both the strategic partners. One-way partner selection techniques available in the literature are impractical as they enable a firm to rank potential partners only from its own perspective while ignoring their objectives. This problem is addressed by the two-way partner selection techniques, however, they either ignore the payoff distribution criteria or the proposed criteria is unfair. More importantly, existing techniques consider that firm collaborate only with the objective to enhance their financial payoff which might not always be the case. The fact that collaborating firms may have one but different objectives for collaboration, or, each may have multiple objectives is largely neglected. To address the scenarios in which firms may collaborate due to multiple and possibly different objectives, this work proposes a bi-objective game-theoretic model that enables a firm to select an appropriate partner based on the individual preferences of both on the following two objectives: 1) learning and 2) financial revenue. Moreover, this model calculates the pay-off that each firm should get whether only monetary, only in the form of learning or both. The calculation of payoff share is based on the following parameters: 1) individual goals of collaboration of partner selecting firms on the said two objectives, 2) their level of cost contribution, 3) cooperation ratio and 4) knowledge investment difference. Comprehensive analysis of various scenarios is done for the proposed Nash Bargaining payoff distribution model to find the optimum strategy of collaborating firms for each scenario.

## Introduction

Technology innovation and acquiring new knowledge is key to the growth of profit of firms in the software industry. With the rapid advancements in technology and added demands of customers, software industry requires to adopt new knowledge to achieve customer satisfaction by improving product quality. With this in mind and the limited resources, it is sometimes difficult to produce a desired quality product in time. Therefore, the best way for a firm to get access to new technologies and develop profitable products is by jointly developing products in strategic alliance with other firms [1].

Strategic alliance can be defined as the cooperative arrangement between two or more independent firms that exchange or share resources for competitive advantage [2]. A strategic alliance may also refer to *“collaborative efforts between two or more firms in which they pool their resources to achieve mutually compatible goals that they could not achieve easily alone”* [3]. A strategic alliance, has various benefits including access to valuable resources [2], capability to rapidly develop high quality products, reduced time to market [4], learning new technologies and trends [1]. In strategic alliance, partners pool their strengths and resources together to achieve their respective goals. These can be to obtain access to new markets, gain knowledge, share risks, technology innovation, improve process performance, quality and productivity [5–9].

Cooperative relation between two firms for a long time based on collaboration is challenging and complex [10]. The behavior and failure of one firm in an alliance may cause alliance to fail [11]. Around 60 percent associations, alliances, joint venture or partnership result in failures [12]. Therefore, selection of an appropriate partner firm is an important factor for success of the alliance, to maximize collaborative profit, and revenue share [13].

Due to rapid technological advancements, requirements for diverse skills and fierce competition in the IT industry, software development firms, especially the new entrants or the smaller ones, find themselves under continuous pressure to gain expertise on diversified skills at the earliest, to become capable enough to develop software products themselves in a timely manner so that they can enhance their financial revenue in future projects. Due to these reasons, such firms may aim to collaborate with those which already have the required expertise. However, a well-established firms may not collaborate with the new entrants as their prime objective is to enhance their financial revenue which they can by forming alliance with equally good or better firms. On the other hand, the prime objective of the new entrants to collaborate is to learn new technologies, skills, and gain access to market. Thus, they might be willing to collaborate even with a lower share of financial revenue as they are getting payoff in the form of learning. On the other hand, a higher share of financial revenue, shall also be acceptable for a well-established firm and as a result both such firms may firm an alliance.

Decision for developing a product in an alliance does not only consider revenue sharing, but also knowledge creation, sharing resources, technological innovation and improving quality and production [6, 9, 14, 15]. The one-way partner selection techniques fail to propose a feasible potential partner firm and/or a fair payoff distribution mechanism. On the other hand, payoff distributions mechanisms based on the two-way partner selection perform better as they consider parameters of both the firms like financial investment, technical expertise, number of invested human resources etc. On the downside, some of them like the model proposed in [13] do consider parameters like cost coordination ratio, knowledge contribution, and learning gained by the collaborative effort. However, the payoff distribution is based solely on the assumption that the firms collaborate only to enhance their financial revenue which as explained above might not always be true; the model proposed in [13] has the same limitations. It does not consider the fact that, in addition to enhancing financial revenue, firms might have multiple objectives for forming a collaboration such as learning new technologies and innovation etc.

Contributions of this Work: Due to the aforementioned prevalent scenarios in the IT industry and limitations in the payoff share calculation mechanisms in the literature, we propose a Nash bargaining based bi-objective game-theoretic model that enables a firm seeking collaboration to select and analyze most suitable partners keeping in view the individual preferences of both the firms on the following two objectives: 1) financial revenue and 2) learning. The proposed model also calculates the payoff share that each of the collaborating firms should get based on their preference over the said two objectives. It is to be noted that two firms may collaborate: a) with only one and the same objective which can either be to increase their financial revenue or the learning payoff, b) with only one but conflicting objectives such as one collaborates only to enhance its financial revenue while the other does so to learn a new technology and innovation, and c) they collaborate to enhance payoff in the form of both the learning and financial revenue with varying preferences over both the objectives. In addition to the individual preferences of the collaborating firms on the said two objectives, the payoff distribution mechanism in our model takes into account the following parameters of the firms: their level of cost contribution, cooperation ratio, knowledge investment, knowledge absorption capacity and trust levels. As these parameters have been very well presented in the model proposed in [13], thus, we adapt these parameters from the same work.

Rest of the paper is organized as follows: section 2 presents an overview of the existing literature on the selection of suitable partner selection. Next, section 3 presents our proposed mathematical model. Furthermore, performance evaluation of our algorithm is presented in section 4. At the end, section 5 concludes this work. The notations used in this paper are presented in the Table 1.

**Table 1:** List of abbreviations.

Notations	Significance
*i*	Knowledge type where *i* = {1…*N*}
*a*	Firm type *a* = {*f*, *p*}
*x*	Initial value of a product
*y*_*i*_	Value added to a product in relation to knowledge type *i*
*v*	Product efficacy
$trust^{a^{{}^{\prime }}a}$	Trust level of a firm *a*′ on *a*
$T_{i}^{a}$	Technical capability of a firm *a* in *i* type of knowledge
$w_{R}^{a}$	Weight of revenue *R* as a goal of firm *a*
$w_{L}^{a}$	Weight of learning *L* as a goal of firm *a*, where $w_{R}^{j}+w_{L}^{j}=1$
$k_{i}^{a}$	Knowledge stock of type *i* of a firm *a*
$\bar{k_{i}}$	Pooled knowledge stock of type *i* of the collaborating firms
$KI_{i}^{a}$	Investment of firm *a* in knowledge type *i*
*β*_*i*_	Knowledge complementarity in knowledge type *i*
*γ*^*a*^()	Knowledge absorption capacity of the firm *a*
*s*()	Coordination cost function
*DC*^*a*^()	Development cost (DC) function of the firm *a*
*θ*	Level of collaboration between firms
*C*	Coordination cost sharing ratio
∅	Revenue sharing ratio

## Literature review

Developing product collaboratively by making alliance has got significant importance in the current era due to access to complementary knowledge, technical abilities, market share. Additionally, collaborative product development improves its quality, increases productivity, and also helps to reduce risks and improve customer satisfaction [1, 2, 4, 14]. However, forming an alliance with a suitable partner is very complex due to involvement of multiple variables, which makes it a multivariate attribute problem. Numerous methods and techniques are proposed to evaluate firms for collaboration formation.

Several techniques have been proposed for the selection of collaboration partner in different industrial domains. The existing partner selection techniques can be broadly classified into one-way partner selection techniques and two-way partner selection techniques.

One way-partner selection techniques enable a firm to rank a list of potential partner firms from the point of view of its own objectives, without considering alliance formation objectives of others. To select partner from single firm perspective several techniques and methods have been proposed in literature; Fuzzy Multiple Attribute Decision- Making (FMADM) for ranking the partner for collaboration formation under complete and incomplete information [8, 16]. Furthermore, applying Formal Concept Analysis (FCA) for evaluation and identification of appropriate partner for horizontal strategic alliance using structural similarities in shipping and manufacturing domain [17]. Similarly, for designing an ideal manufacturing chain, [18] uses practical swarm optimization (PSO) along with learning scheme. Furthermore, [5] uses intuitionistic fuzzy for project partner selection. Similarly applying vague set theories to evaluate partner under uncertain information [19]. However, Multi-objective programming model (MOP) is also used for selecting partner but keeping in view the objectives of firm such as customer satisfaction, quality and to maximize profit [2]. Another technique, analytic network process (ANP) is used for ranking potential candidates considering multivariate attributes [20]. Moreover, Bayesian network (BN) was applied for selection of successful collaborative partner based on firms technological capabilities [21].

From the above discussion, one can observe that most of the existing techniques focus on the partner selection problem. However, they are limited to selection of partner from the perspective of one firm which is assessing other potential partner firms based on its own goals without considering goals or objectives of the others. The best potential partner suggested by such techniques may never be interested in forming collaboration making such solutions impractical. For instance, it may happen that firm A is the best partner choice for firm B but vice versa may not be true. Realizing this limitation, [6] proposed a partner selection technique which enables potential partnership seekers to also evaluate each other resulting in better outcomes. However, once the partners collaborate it is unclear how profits should be distributed among collaborating firms.

In reality, firms are rational agents interacting in a strategic situation in the sense that everything they do is to maximize their payoff and the payoff that each gets is interdependent on the actions of each other. Game theory is an effective mathematical modelling tool to formulate, analyze and solve strategic situations like collaboration formation between firms and revenue sharing decision between them. It has already been used to solve numerous collaboration related problems in various domains as shown in Table 2. For instance, for revenue sharing problem in collaboration domain, [14] used Nash Bargaining approach to determine the level of investment, revenue and innovation sharing. Although, this work provides different sharing ratios among firms while forming collaboration but this approach is only applicable where firms have similar agenda towards collaboration. Furthermore, [22, 23] applied Stackelberg leader-follower game for investigating revenue sharing mechanism in reverse logistic and R&D alliance. In general the focus of these works is to form alliance among two firms, however, they focus only on a single goal that is to increase performance using revenue sharing mechanism. Moreover, [24] provides Nash solution on cost and profit sharing in research and development alliance by considering the profit sharing with equal distribution of power. Although, this work is effective from managerial perspective but it is also limited to two firms. In addition, this work also takes into account the similar agenda of firms towards collaboration. Similarly, Nash bargaining solution was used to analyze the various conditions under which collaboration can be developed to decide the level of investment, collaboration and revenue share in [13]. Furthermore, this study also explores the co-innovation and co-learning dimensions for collaborative product development. Likewise, this work also neglects the fact that different perspectives of firms towards collaboration may affect both the decisions for forming it and the revenue sharing criteria as well. A summary of game theoretic techniques relevant to collaboration is presented in the Table 2.

**Table 2:** Game theory literature on collaboration.

Domain	Focus	Approach	Findings	Factors	Ref.
Collaborative product development	Collaboration formation and revenue sharing decision	Nash Bargaining	The model can help collaborating parties in negotiation on revenue sharing	Innovation, co-learning, trust, knowledge investment, product efficacy, knowledge complementarity	[13]
Collaborative product development	Innovation sharing, investment sharing and revenue sharing	Nash bargaining	Providing guidelines and strategies of level of innovation, Investment and revenue sharing	investment, innovation, technology	[14]
Supply chains	Reverse logistic revenue sharing decision	Stackelberg leader-follower	Achieve higher environmental performance	Performance	[22]
R& D alliance	Decision on ROR (rate of return)	Stackelberg leader-follower	Providing strategy for resolving conflicts of optimal ROR (rate of return)	Innovation, Risk, quality	[23]
R& D alliance	Asymmetric and Symmetric distribution of power between firms	Nash equilibrium	Equal distribution of power maximize research effort	Effort, investment, knowledge creation, performance	[24]
Supply chains	Revenue sharing and product substitution decision	Nash equilibrium	Provide guidance on managing relation between two manufacturers and one retailer in supply chain	Performance, market, productivity	[25]
E-collaboration	Decision of collaboration formation and Level of collaboration	Snow drift and prisoners dilemma	preferences may change the expected payoff	Collaboration level	[26]
Strategic alliance	Behaviour effect in same-functional alliance vs cross sectional alliance	Nash equilibrium	Same functional alliance perform well compared to cross-functional alliances when all else are equal	Learning, effort, investment	[27]
E-collaboration	Collaborative learning	Evolutionary game theory	Low and negligible perception is the major reason for decision to collaborate	Learning	[28]
Collaborative learning	Competition based learning	Prisoners dilemma	Completion based learning can help to adopt diverse learning styles	Learning, performance	[29]

To summarize the aforementioned game theoretic techniques on revenue sharing and collaboration formation we concluded that, existing game theoretic based collaboration formation techniques ignore the fact that firms may have different and at the same time possibly multiple objectives towards forming collaboration. That is one firm may prefer to attain knowledge in the first priority and other collaborating firm may prefer monetary profit, innovation or any other goal. Alliances are built based on agenda of the firms, Therefore, it is necessary that the decision on profit sharing reflects the objectives of firms towards collaboration. Our model reflects the situation where firms decision towards collaboration formation and profit sharing is based on both the monetary revenue and learning.

In this work, we extend the frame work proposed by [13]; our model enables a firm to select partners based on two objectives i.e. learning and financial revenue. Our Nash bargaining based game theoretic model calculates the payoff that collaborating firms should get based on their preferences of forming alliance on the said two objectives i.e. learning and monetary profit, their level of cost contribution, cooperation ratio and knowledge investment. As stated above, this model also focuses on revenue division while considering goals of partnering firms for forming collaboration. Firms can have same or conflicting goals; one firm may prefer learning a new technology and innovation while the other may be interested only in enhancing financial revenue. It is also possible that a firm may collaboration with the aim to enhance its payoff in terms of both the financial revenue and learning. In the next section, we present our Nash Bargaining based game theoretic model.

## Proposed model

In this section, we model the situation where firms can have same or conflicting goals from alliance. The model will provide optimal solution for revenue sharing. In our model two firms are considered for collaboration namely focal (*f*) and partner (*p*). Moreover, firms consider two goals i.e. revenue (*R*) and learning (*L*) for forming alliance. The profit utility function consists of both the revenue and learning. A firm may have: a) only one of the two mentioned objectives to collaborate, b) both the objectives while having equal or different preference over them.

To provide more realistic model related to revenue sharing between two firms (*f*) and (*p*), we considered the model proposed in [13] and incorporate the concepts of multiple objectives such as learning and revenue. To calculate the final revenue sharing between firms (*f*) and (*p*), we develop the model in steps. First, we formulate the stock of knowledge for a firm in terms of *a* and *a*′. In the second step, we derive the payoff of firms from learning objective. Next, we utilize the leaning and stock of knowledge to formulate co-learning and pooled stock of knowledge. In the next step, we use the pooled stock of knowledge to formulate payoff functions for firm *f* and *p*. Finally, we formulate the revenue share as Nash Bargaining problem.

**Stock Of Knowledge a Firm:** Firms having high stock of knowledge ($k_{i}^{a}$) have the capability to develop product effectively and efficiently resulting in increased profit. However, firms with low stock of knowledge can collaborate with another firm to increase its knowledge stock of knowledge. Therefore, in strategic alliance firm’s having different capability collaborate and pool their resources to gain new knowledge that knowledge can be used as input to new projects. The stock of *i* type of knowledge for firm *a* can be defined as

$$\begin{array}{c} k_{i}^{a}={KI}_{i}^{a}+\theta \gamma^{a}\left({KI}_{i}^{a},\beta_{i}\right){\left({KI}_{i}^{a^{{}^{\prime }}}T_{i}^{a^{{}^{\prime }}}\right)}^{{trust}^{a^{{}^{\prime }}a}} \end{array}$$ (1)

Firms can have *N* different type of knowledge [30] whereas *a* and *a*′ represents *f* and *p* firm or vice versa. $\gamma^{a}\left({KI}_{i}^{a},\beta_{i}\right)$ is the knowledge absorption function and is discussed later. The stock of knowledge for firm *a* is the result of firms own investment $KI_{i}^{a}$ in *i* type of knowledge and the knowledge absorbed in collaboration with *a*′. Where *a*′ represent the collaborating firm. Here *a* and *a*′ can be considered as f and p firms or vise verse.

**Payoff from Learning:** In collaboration, firms stock of knowledge $k_{i}^{a}$ is resultant of its own investment and knowledge absorbed while collaborating with another firm as shown in Eq 1. Therefore, we considers the the abortion part as the learning ($\pi_{L}^{a}$) for a single firm as shown bellow

$$\begin{array}{c} \pi_{L}^{a}=\theta \gamma^{a}\left({KI}_{i}^{a},\beta_{i}\right){\left({KI}_{i}^{a^{{}^{\prime }}}T_{i}^{a^{{}^{\prime }}}\right)}^{{trust}^{a^{{}^{\prime }}a}} \end{array}$$ (2)

Eq 2 represents the learning payoff of a firm *a* (*f* or *p*). Trust plays important role in sharing of knowledge as high trust among firms ensures greater learning. Therefore ${trust}^{a^{{}^{\prime }}a}$ represents the trust of firm *a*′ on *a*. Trust is the important factor and has positive effect on the alliance collaboration [13][31][32][33]. Technical ability of a firm shows the level of knowledge it possesses in knowledge type *i*. Investment of a firm is also a major factor for collaboration [34]. Partner firms investment and technical ability can only be truly revealed to the focal firm if they have high trust on each other and the level of collaboration (*θ*) between firms is high [13]. Moreover, absorption capacity function *γ*^*a*^ of firm *a* is the combination of its own investment and the level of knowledge difference between firms *a* (*f* or *p*) and *a*′. High stock of knowledge can be attained by firm *a* when *a*′ has high technical level in *i* type of knowledge.

**Co-learning:** As we discussed the learning of a single firm in Eq 2. We can use it to formulate the co-learning function for firm *f* and *p*. Therefore, considering collaboration between firms *f* and *p*, the co-learning for both of them will be then represented as the sum of leaning of firm *f* and *p* as follows:

$$\begin{array}{c} \theta [\gamma^{f}\left({KI}_{i}^{f},\beta_{i}\right){\left({KI}_{i}^{p}T_{i}^{p}\right)}^{{trust}^{pf}}+\gamma^{p}\left({KI}_{i}^{p},\beta_{i}\right){\left({KI}_{i}^{f}T_{i}^{f}\right)}^{{trust}^{fp}}] \end{array}$$ (3)

**Pooled Stock Of Knowledge:** While collaborating firms develop product collaboratively. For this reason it is necessary to combine their stocks of knowledge. We called the combined stock of knowledge as pooled stock of knowledge (${\bar{k}}_{i}$). Therefore, considering Eq 1, the pooled stock of *i* type of knowledge becomes the sum of investment of both firms and the knowledge added by co-learning, and can be written as:

$$\begin{array}{c} \begin{array}{c} {\bar{k}}_{i}={KI}_{i}^{f}+{KI}_{i}^{p}+\theta [\gamma^{f}\left({KI}_{i}^{f},\beta_{i}\right){\left({KI}_{i}^{p}T_{i}^{p}\right)}^{{trust}^{pf}}\\ +\gamma^{p}\left({KI}_{i}^{p},\beta_{i}\right){\left({KI}_{i}^{f}T_{i}^{f}\right)}^{{trust}^{fp}}] \end{array} \end{array}$$ (4)

**Payoffs When Firms Collaborate:** We utilize the stock of knowledge to formulate the payoff equations for firm *f* and *p*. Indeed, the stock of knowledge has direct effect on the value added to the product. It is uncertain whether the new knowledge can improve the final value added to the product. Literature refers to this uncertainty as transitional uncertainty, *$y\bar{k}\bar{v}$* is the final value added to the product if we represent the efficacy of product as *$\bar{v}$* as shown in Eqs 5 and 6. The value of *v* ranges from 0 to 1. As the value of *v* increases, a firm faces less uncertainty in developing valuable product [14].

In a revenue sharing mechanism if focal firm (*f*) gets ∅ then the partner firm (*p*) gets 1 − ∅. In collaboration, the cost borne by a firm is the cost due to coordination, its own internal development cost and knowledge investment cost [13] as shown in Eqs 5 and 6.

Coordination cost ((1 − *C*)*s*(*θ*)) is dependant on the level of collaboration; the higher the collaboration level the higher the coordination cost will be. Collaboration level is the willingness of a firm to share information and reveal its technical abilities to another firm. The coordination cost occurs because of the collaboration. For this reason, the coordination cost is shared by firms. In terms of ratio, firm *f* bears cost *1* − *C* and *p* bears *C*.

$KI_{i}^{a}$ represents the knowledge investment by firm *a* (*f* or *p*). So the total knowledge investment cost born by firm *a* will be $\sum_{i}{KI}_{i}^{a}$. The development cost is associated with the stock of knowledge created by collaboration and investment by firms; the higher the investment and stock of knowledge lesser will be the development cost. It is to be noted that the development cost always exists even when stock of knowledge or investment are both high [13].

Thus, concluding from the above discussions the payoff function of focal and partner firms in terms of revenue are [13]:

$$\begin{array}{c} \pi_{R}^{f}=\emptyset \left(x+y\bar{k}\bar{v}\right)-\left(1-C\right)s\left(\theta \right)-\sum_{i=1}^{N}\left({KI}_{i}^{f}+DC_{i}^{f}\left(k_{i}^{f}\right)\right) \end{array}$$ (5)

And

$$\begin{array}{c} \pi_{R}^{p}=\left(1-\emptyset \right)\left(x+y\bar{k}\bar{v}\right)-Cs\left(\theta \right)-\sum_{i=1}^{N}\left({KI}_{i}^{p}+DC_{i}^{p}\left(k_{i}^{p}\right)\right) \end{array}$$ (6)

Therefore, the expected total profit terms of revenue and learning for *f* and *p* firm is as follow.

$$\begin{array}{c} \begin{array}{c} \pi_{tot}^{f}=[\emptyset \left(x+y\bar{k}\left(\frac{v+1}{2}\right)\right)-\left(1-C\right)s\left(\theta \right)-\\ \sum_{i=1}^{N}\left({KI}_{i}^{f}+DC_{i}^{f}\left(k_{i}^{f}\right)\right)]+\left[\theta \gamma^{f}\left({KI}_{i}^{f},\beta_{i}\right){\left({KI}_{i}^{p}T_{i}^{p}\right)}^{{trust}^{pf}}\right] \end{array} \end{array}$$ (7)

And

$$\begin{array}{c} \begin{array}{c} \pi_{tot}^{p}=[\left(1-\emptyset \right)\left(x+y\bar{k}\left(\frac{v+1}{2}\right)\right)-Cs\left(\theta \right)-\\ \sum_{i=1}^{N}\left({KI}_{i}^{p}+{DC}_{i}^{p}\left(k_{i}^{p}\right)\right)]+\left[\theta \gamma^{p}\left({KI}_{i}^{p},\beta_{i}\right){\left({KI}_{i}^{f}T_{i}^{f}\right)}^{{trust}^{fp}}\right] \end{array} \end{array}$$ (8)

**Writing Payoffs In a Compact Way:** The absorption capacity function of firm *a* (*f* or *p*) is $\gamma^{a}\left({KI}_{i}^{a},\beta_{i}\right)=KI_{i}^{a}\beta_{i}+\left(1-\beta_{i}\right)$. Therefore, the learning for a firm *a* will become $L^{a}=\left(KI_{i}^{a}\beta_{i}+\left(1-\beta_{i}\right)\right){\left(KI_{i}^{a^{{}^{\prime }}}T_{i}^{a^{{}^{\prime }}}\right)}^{{trust}^{a^{{}^{\prime }}a}}$. Hence the co learning for *f* and *p* firm will be then *θ*(*L*^*f*^+ *L*^*p*^). With this consideration of co-learning the total pooled stock of knowledge for *f* and *p* firms become $k_{i}^{a}=KI_{i}^{f}+KI_{i}^{p}+\left(L^{f}+L^{p}\right)$. Similarly, the payoff function of learning for firm *a* (*f* or *p*) will be then $\pi_{L}^{a}=\theta L^{a}$. The development cost function depends on initial development cost *DC* and development cost decrease rate *DCDR* in relation to the knowledge investment and learning of firm *a* and will be expressed as *DC*^*a*^ − *DCDR*^*a*^(*KI*^*a*^+ *θL*^*a*^) [13]. The coordination cost function in relation to *θ* can be expressed as *s*(*θ*) = *I*^2^ where *I* is the associated investment cost. Finally, introducing some new notations $\bar{x}=x+y(\left(\frac{v+1}{2}\right)\left(KI^{f}+KI^{p}\right),\bar{y}=y(\left(\frac{v+1}{2}\right)\left(L^{f}+L^{p}\right),\bar{DC^{a}}=DC^{a}+\left(1-{DCDR}^{a}\right)\left(KI^{a}\right)$ and $\bar{{DCDR}^{a}}={DCDR}^{a}L^{a}$ where *a* can be *f* or *p* firm. Therefore, using compact notations we rewrite Eqs 7 and 8 as follow

$$\begin{array}{c} \pi_{tot}^{f}=\emptyset \left(\bar{x}+\bar{y}\theta \right)\left(1-C\right)I\theta^{2}-\left(\bar{DC^{f}}-\bar{{DCDR}^{f}}\theta \right)+\theta L^{f} \end{array}$$ (9)

And

$$\begin{array}{c} \pi_{tot}^{p}=\left(1-\emptyset \right)\left(\bar{x}+\bar{y}\theta \right)-CI\theta^{2}-\left(\bar{DC^{p}}-\bar{{DCDR}^{p}}\theta \right)+\theta L^{p} \end{array}$$ (10)

The total utility function for focal and partner firms along with goals of firms will be

$$\begin{array}{c} \pi_{tot}^{f}=w_{R}^{f}\pi_{R}^{f}+w_{L}^{f}\pi_{L}^{f} \end{array}$$ (11)

$$\begin{array}{c} \pi_{tot}^{p}=w_{R}^{p}\pi_{R}^{p}+w_{L}^{p}\pi_{L}^{p} \end{array}$$ (12)

Where $w_{R}^{f}$ and $w_{L}^{f}$ are the weights assigned to the revenue and learning goals respectively by focal (*f*) firm. Whereas $w_{R}^{p}$ and $w_{L}^{p}$ are the weights assigned to the revenue and learning goals respectively by partner (*p*) firm. The revenue that each firm gets from collaboration depend on their preference toward the goals. The weights are assigned in a linguistic terms for both goals and the value of weights ranges from 0 to 1. Note that the sum of the weights assigned to both goals by a single firm should be equal to 1.

**Nash Bargaining Problem Solution:** A three stage solution is presented for finding Nash bargaining solution for our proposed model as done in [13]. In the first stage, focal firm decides the level of collaboration *θ* and the collaboration level is in accordance with its requirements. The optimal θ* is calculated through utility function of focal firm given in Eq 9.

$$\begin{array}{c} \theta^{*}=-\frac{\left(\bar{y}\emptyset +L^{f}+\bar{{DCDR}^{f}}\right)}{\left(2IC-2I\right)} \end{array}$$ (13)

Proof of the equation cannot be provided here due to its lengthiness. However, in order to calculate optimal collaboration level θ*, we take the first order derivative of the utility function of the focal firm provided in the Eq 9, and then solve it for θ*. Optimal θ* is the level of information shared among firms and is derived from the payoff function of the focal firm. The greater the collaboration level the higher the productivity will be, whereas, higher collaboration level increases the coordination cost as well. Therefore, in order to generate higher payoff, it is necessary to calculate an optimal collaboration level as it affects the coordination cost ratio decision which ultimately can result in a high or a low payoff. In the second stage, the optimal value of θ* is inserted in the profit function of the partner firm in the Eq 10, and then we take its first order derivative to calculate an optimal coordination cost sharing ratio *C*. As a result, we get:

$$\begin{array}{c} C=-\frac{2L^{p}-3\bar{y}\emptyset +2\bar{{DCDR}^{p}}-L^{f}-\bar{{DCDR}^{f}}+2\bar{y}}{2L^{p}-\bar{y}\emptyset +2\bar{{DCDR}^{p}}+L^{f}+\bar{{DCDR}^{f}}+2\bar{y}} \end{array}$$ (14)

Before moving to the next step, we put the optimal θ* and optimal coordination cost ratio *C* derived above in the final payoff functions of both the focal and the partner firms. Due to lengthiness of the proof of equations, it is not provided here. In the final stage, the optimal revenue sharing level ∅* is calculated by solving the Nash bargaining as a revenue maximization problem.

$$\begin{array}{c} \pi_{tot}^{f}*\pi_{tot}^{p} \end{array}$$ (15)

Optimal ∅* is very complex to provide in this paper. However, the optimal ∅* is derived by taking first order derivative of Eq 15.

In this section, we formulated the strategic interaction between two potential partner firms as a bi-objective Nash Bargaining game theoretic model. The proposed model calculates the payoff share of both the focal and the partner firms based on their level of preferences over the two objectives: a) learning and b) financial revenue. The proposed model takes into account the cost coordination ratio, knowledge investment, knowledge absorption capacity, trust level and efficacy. Using the total payoff functions, we derived optimal collaboration level (θ*) and optimal coordination cost ratio (*C*) for the firms. In final stage, we used θ* and *C* to derive the optimal revenue share (∅*) as a Nash Bargaining problem. The resultant ∅* will reflect the share of revenue based on the objectives of the firms.

In the next section, we extensively evaluate the performance of our proposed model in numerous scenarios.

## Performance evaluation

Performance of the proposed game-theoretic bi-objective collaboration formation model is evaluated via extensive simulations. We study the impact of the following four parameters on the total payoff that the product developed jointly generates and the revenue sharing ratio of firms: 1) Objective of the firms for forming collaboration, 2) knowledge investment, 3) trust level of firms on each other and 4) efficacy and product type. By adopting the different scenarios provided in [13] which are recognized by the industrial expert of The Scientific and Technological Research Council of Turkey (TUBITAK) and adding additional parameters to the scenario legend such as technical capability of a firm in knowledge type *i* and the preferences of a firm towards collaboration is presented in the Table 3.

**Table 3:** Scenario legend.

Fig	Trust	Knowledge Investment	Initial Dc	DCDR	Product type	Technical level	Product efficacy	Goals preference
1	High and equal	High and equal	Equal	Low and equal	New	High and equal	Moderate	***Varying***
2	High and equal	F High	Equal	Low and equal	New	High and equal	Moderate	
	High and equal	P High	Equal	Low and equal	New	High and equal	Moderate	
	High and equal	F High	Equal	Low and equal	New	F High	Moderate	
	High and equal	P High	Equal	Low and equal	New	P High	Moderate	
3	High and equal	***P high F Varying***	Equal	low and equal	New	High and equal	Moderate	Equal
	P High		*F* > *P*	Low and equal	New	F High	Moderate	Equal
	High and equal		Equal	P High	New	F High	Moderate	Equal
4	***Varying***	High and equal	Equal	Low and equal	New	High and equal	Moderate	Equal
		P Higher	Equal	Low and equal	New	F High	Moderate	Equal
		F Higher	Equal	Low and equal	New	F High	Moderate	P prefer revenue
5	High and equal	Moderate and equal	Equal	High and equal	New	Moderate equal	low	Equal
	High and equal	Moderate and equal	Equal	High and equal	New	Moderate equal	Moderate	Equal
	High and equal	Moderate and equal	Equal	High and equal	Upgrade	Moderate equal	low	Equal
	High and equal	Moderate and equal	Equal	High and equal	Upgrade	Moderate equal	Moderate	Equal
	High and equal	Moderate and equal	Equal	High and equal	New	Moderate equal	High	Equal
	High and equal	Moderate and equal	Equal	High and equal	Upgrade	Moderate equal	High	Equal

Unless otherwise specified, the x-axis in figures represents revenue sharing ratio of the focal firm *f*, and the y-axis represents its total profit change in relation to the revenue sharing ratio. The values of all the considered parameters are normalized on the scale of 0-1 in accordance with the scenarios considered in [13]. The ranges are specified in linguistic terms such as for low, moderate and high we assigned the ranges from 0 or 0.1 to 0.3, 0.31 to 0.6 and 0.6 to 1 respectively. Following subsections demonstrate the impact of different parameters on total profit and revenue sharing decision.

### Impact of variation in preferences of firms

**Fig 1:**
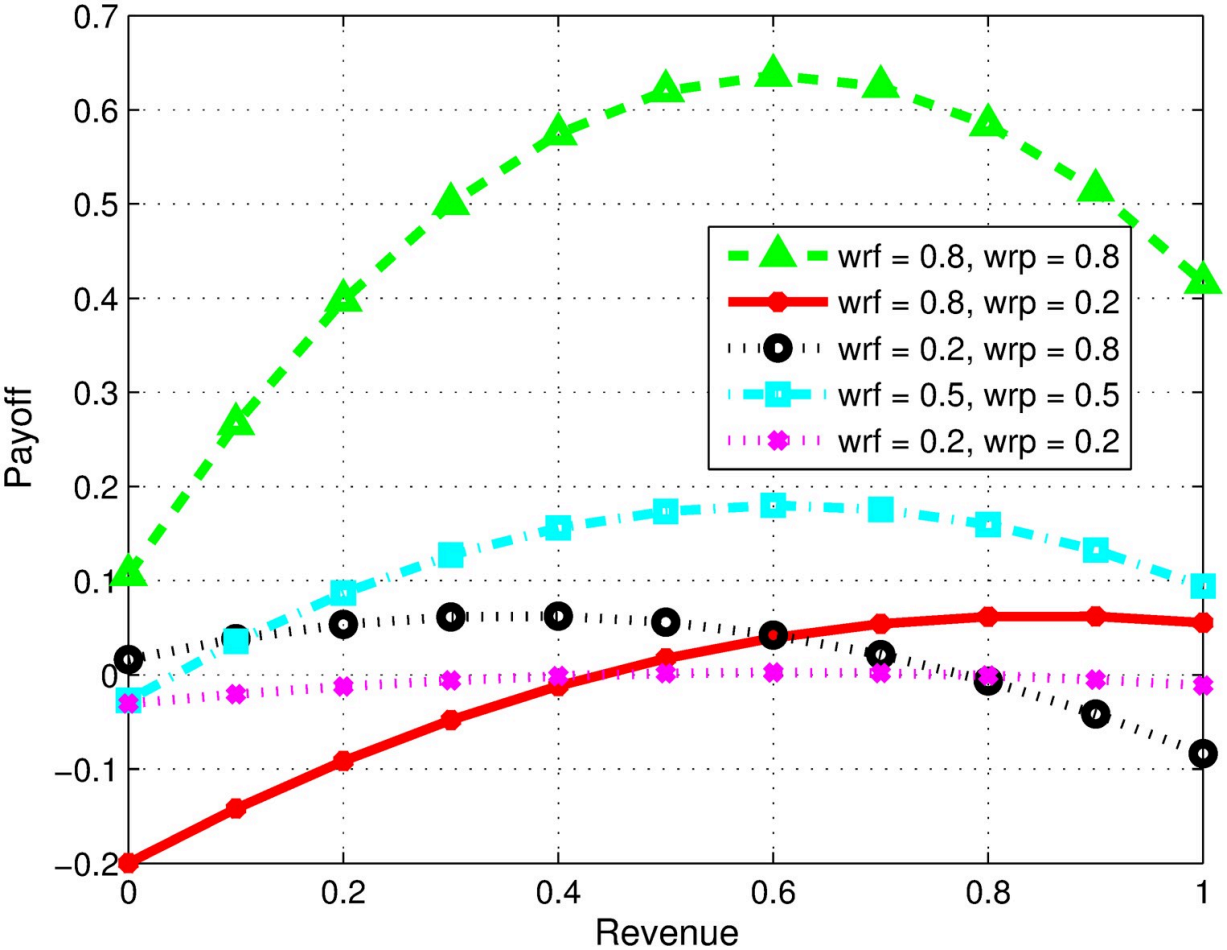
Impact of variation in preferences of firms.

**Fig 2:**
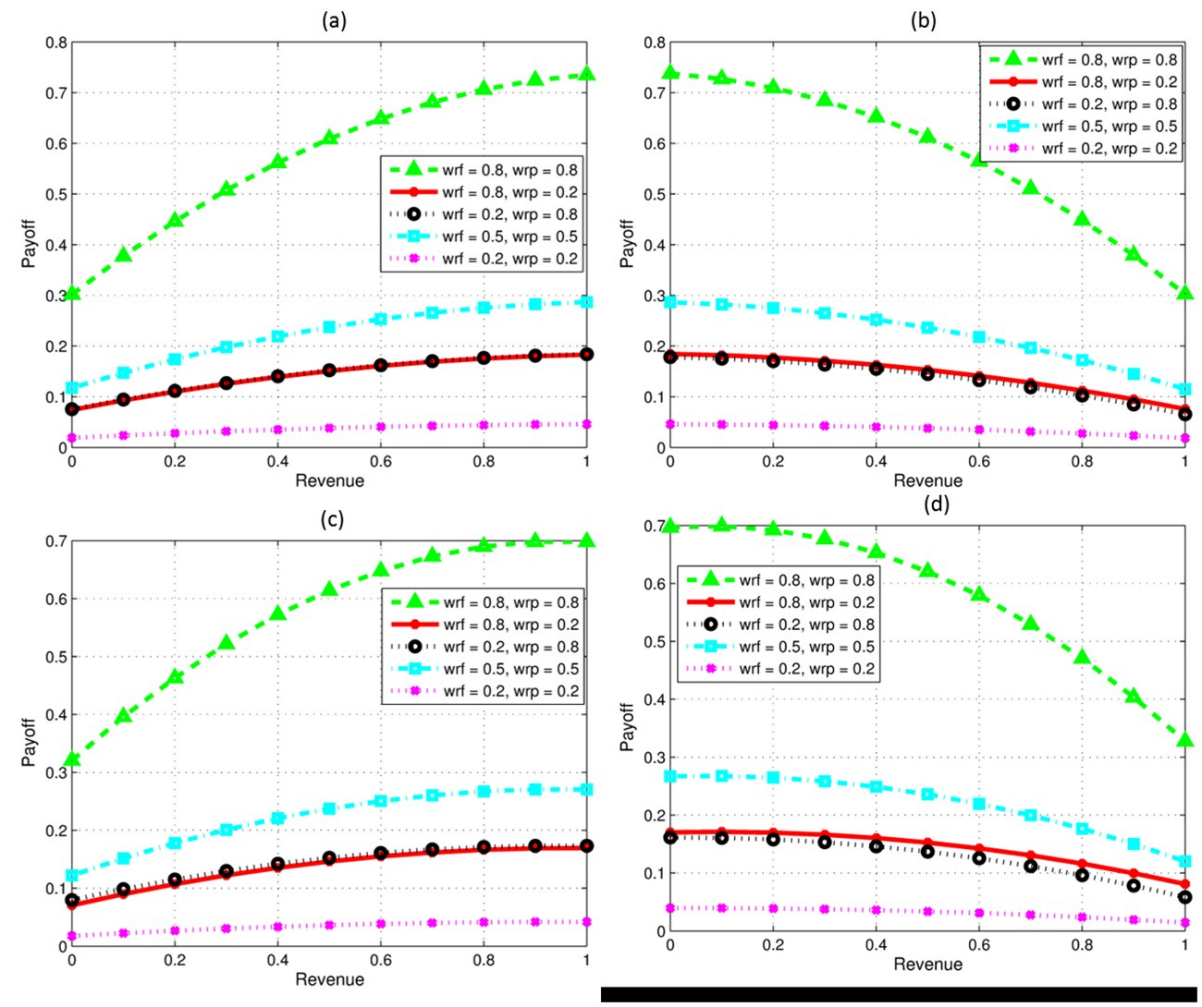
Impact of the individual objectives of firms over financial revenue and learning payoff. (a) F has high knowledge investment. (b) Fig 2b. P has high knowledge investment. (c) F has high investment and technical skills. (d) P has high investment and technical skills.

In our model, firms consider two goals i.e. learning and revenue while forming collaboration. Here, we study the impact of preferences of firms. Fig 1 reflects the situation where firms are equal in their capabilities but can have the same or conflicting goals towards alliance. The changing preferences are analyzed for revenue sharing decision. In Figs 1 and 2, the terms *wrf* and *wrp* represent the preference level of focal and partner firms towards monetary revenue respectively. In Fig 1, the first curve represents payoff share of focal firm, when both partners prefer high monetary revenue compared to learning. In this case, the optimal revenue sharing ratio is 0.5 for a given scenario as both firms have equal competencies other than their perspectives. However, the second curve represents payoff ratio, when *f* prefers high monetary revenue, whereas, the partner firm highly prefers learning to monetary revenue. In this case, higher collaborative profit can be obtained if partner firm gets less revenue share, keeping in view that both firms are similar by all means other than their preferences. Similarly, the third curve represents payoff share when the focal firm prefers learning more and partner firm prefers revenue more. To achieve high collaborative profit, the focal firm in this scenario should agree on a lesser monetary revenue share. The fourth curve represents the payoffs when both firms prefer financial revenue and learning equally. Therefore, in order to achieve a higher profit the model suggests that the both shall have equal share of revenue. Similarly, the last curve represents with both firms prefers learning instead of revenue and the results suggests that both firm shall agree on equal revenue share.

Fig (2a) shows the results when only the knowledge investment of focal firm is higher, and all other capabilities of both the firms are same. The collaborative profit will be maximum if partner firm settles on a strictly lesser revenue share than *f*, irrespective of whether the preferences of both the firms are same or conflicting. Contrarily, Fig (2b) represents scenario where partner firm P invests higher compared to focal firm; the decision will be opposite to scenario presented in Fig (2a) where collaborative profit will be high if p gets most of the revenue share. Next, Fig (2c) represents the case when *f* has high knowledge investment and high technical skills. As shown, the collaborative profit will be high if *f* gets almost all revenue regardless of whether both the firms have same or conflicting perspectives towards collaboration. Similarly, Fig (2d) represents the scenario where *p* has comparatively both high knowledge investment and high technical skills. For obtaining high collaborative profit the optimal solution on revenue share will be for P to get high revenue share regardless of perspectives of both the firms for collaboration.

### Impact of knowledge investment

**Fig 3:**
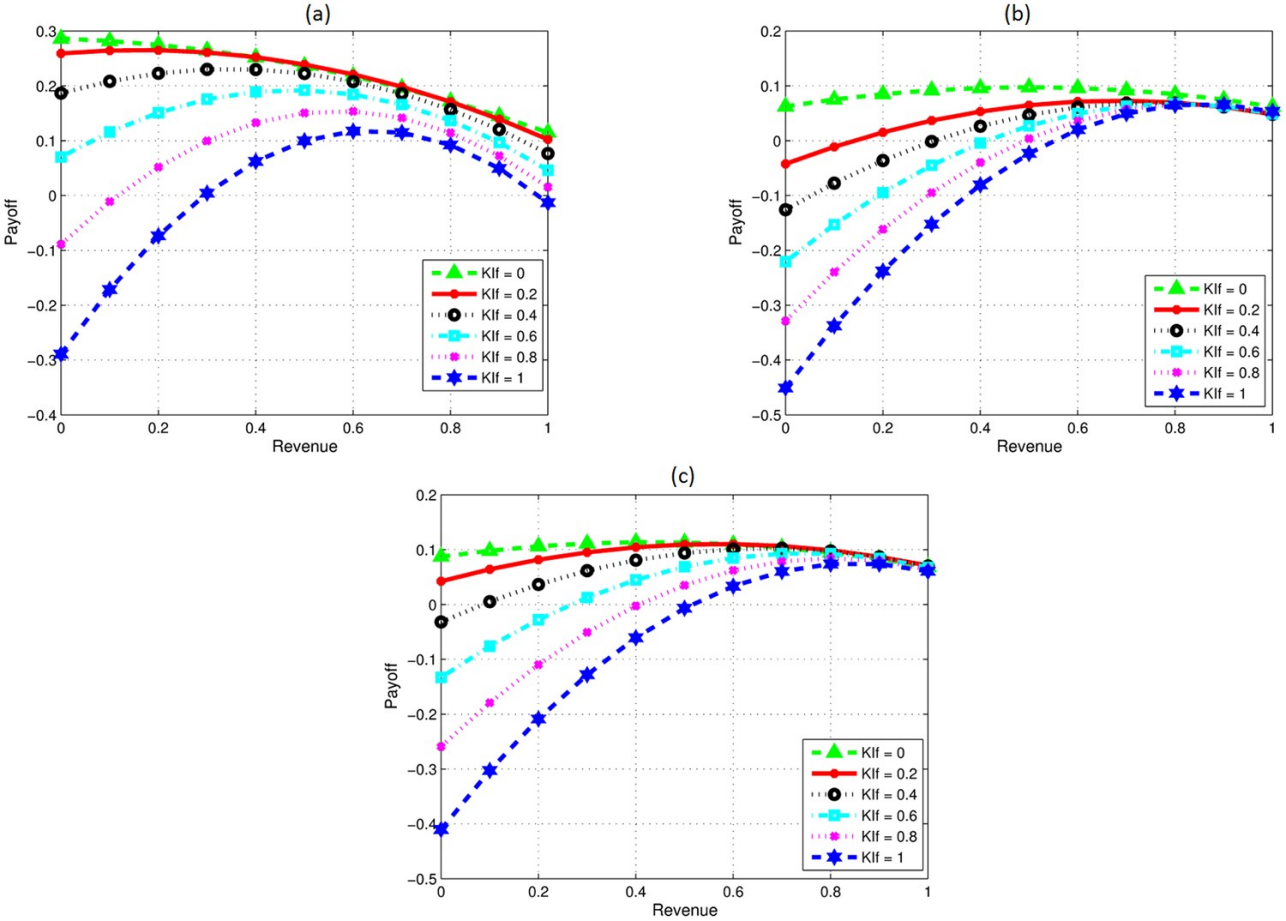
Impact of varying knowledge investment by F. (a) P high F varying investment. (b) High initial DC and technical skills for F. (c) DCDR for P high, F high technical skills.

Knowledge investment is an important factor of collaboration as it effects learning and revenue of the firms. In this section, we study the impact of knowledge investment level on the revenue sharing ratio and overall payoff. We consider the scenarios where the knowledge investment by *p* is higher initially, and it varies for focal firm. In Fig 3, KIf represents the varying knowledge investments by the focal firm.

Considering new product development along with variation in knowledge investment is shown in Fig (3a). Results show that the firm investing higher gets more profit share. The decision on revenue sharing changes if we increase the knowledge investment by focal firm gradually. The optimal solution for each investment level is different. With less investment by focal firm, the optimal revenue sharing decision for it is to settle on less revenue share. As the investment by *f* increases, higher collaborative profit can be obtained when both firms get equal share of revenue due to the fact that both firms become equal in all aspects.

Similarly in Fig (3b), we consider again the case when firms collaborate for developing a new product development. Again, we vary the knowledge investment by *f*. However, we consider the case where *f* pays higher initial development cost than *p*, and also possesses higher technical abilities. Results show that the collaborative profit will be high, if *f* gets most of the revenue share even with less investment as it adds more value to the product with its better technical abilities. Optimal solution for revenue share is 0.5 initially when the focal firm invests nothing. However, it gradually changes with the increase in investment by *f*.

Results for new product development with higher development cost decrease rate (DCDR) by the partner firm and high technical ability with varying knowledge investment by focal firm are presented in Fig (3c). Optimal revenue share decision varies with increasing investment level by focal firms. With no or less investment by *f*, the optimal revenue share is to have equal division. This is due to the fact that one firm invests higher and the other has high technical skill. However, the optimal revenue share decision changes with increase in investment by *f*, and the collaborative profit will be maximum when *f* gets most of the revenue.

### Impact of trust level

**Fig 4:**
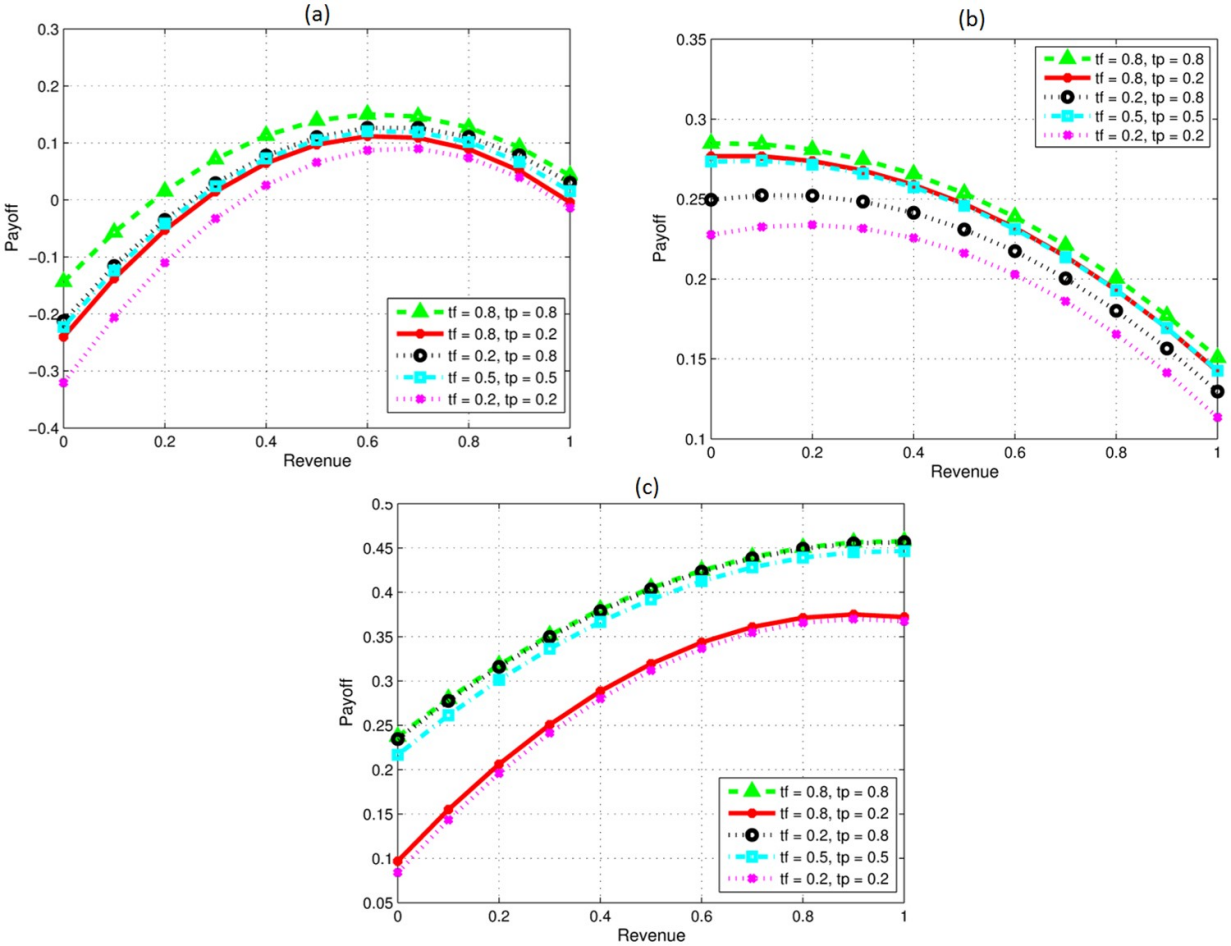
Impact of trust level. (a) Varying trust only. (b) P high knowledge investment, F high technical skills. (c) Trust varying, P high knowledge investment and high technical skills.

Trust is an important factor for successful collaboration as mentioned in the literature [33][13]. Level of trust (tf) shows the willingness of a firm to share information with its partner. In Fig 4, we evaluate the impact of trust between firms on the payoff and revenue share for various scenarios. In Fig (4a), development of a new product is considered. Results show that the profit is high when both firms have high level mutual trust, and it decreases for lower trust levels. However trust level, whether low, high, equal or unequal, does not affect the revenue sharing decision of firms. High trust reflects that firms share information and their resources to high extent resulting in higher profit, whereas, low trust level restrict firms from sharing information and resources resulting in low profit. Therefore, high or low trust levels may not affect the revenue sharing decision when all other parameters of the firms are equal. Whereas, with different capability (parameters) levels the revenue sharing decision may be affected.

Fig (4b) presents the scenario where *p* has higher investment and *f* has high technical skills. In this situation, the firm with higher investment gets most of the revenue no matter what technical abilities other partner has if collaborative profit is to be high for all level of trust. Similarly in Fig (4c), *f* gets most of the revenue share as its knowledge investment and technical ability has major role in the overall profit regardless of the preferences of both the firms towards collaboration and the level of trust between them.

### Impact of efficacy and product type

**Fig 5:**
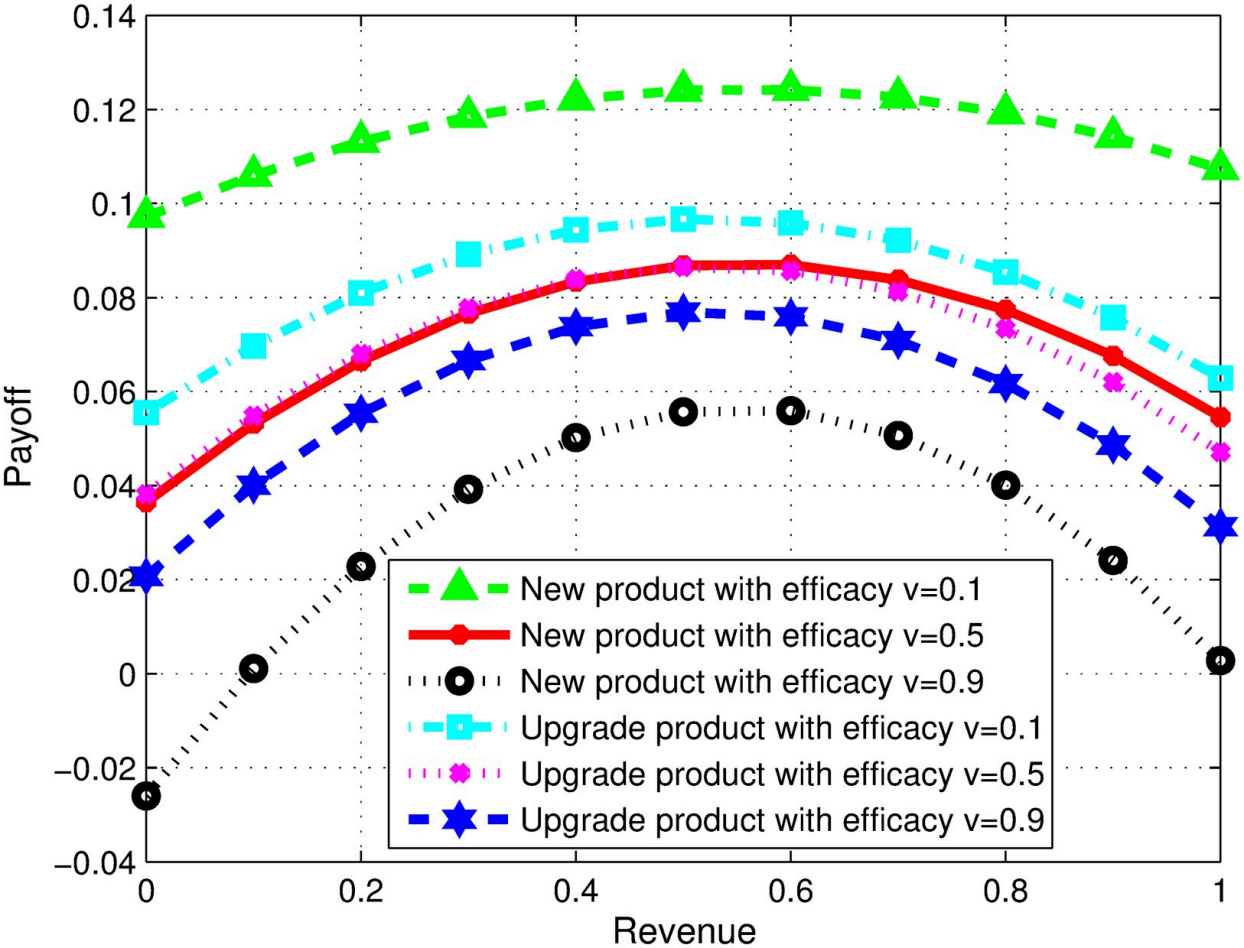
Impact of efficacy and product type.

Efficacy of collaborating firms plays an important role in the success of collaboration and thus impacts overall profit. In addition, whether firms collaborate to develop a new product or to improve an existing one also impacts the total payoff. This section evaluates the impact of developing a new product or adding value to an existing one in combination with low, moderate and high efficacy on the revenue sharing ratio and total generated payoff. In Fig 5, four scenarios are presented. The total profit in all cases differs but the optimal revenue sharing ratio in each case is 0.5 for new product development as well as for product upgrades. The product type may affect the profit but not the revenue decision considering firms with similar abilities. This is because all other parameters for both the firms are considered to be same.

### Comparative analysis

**Fig 6:**
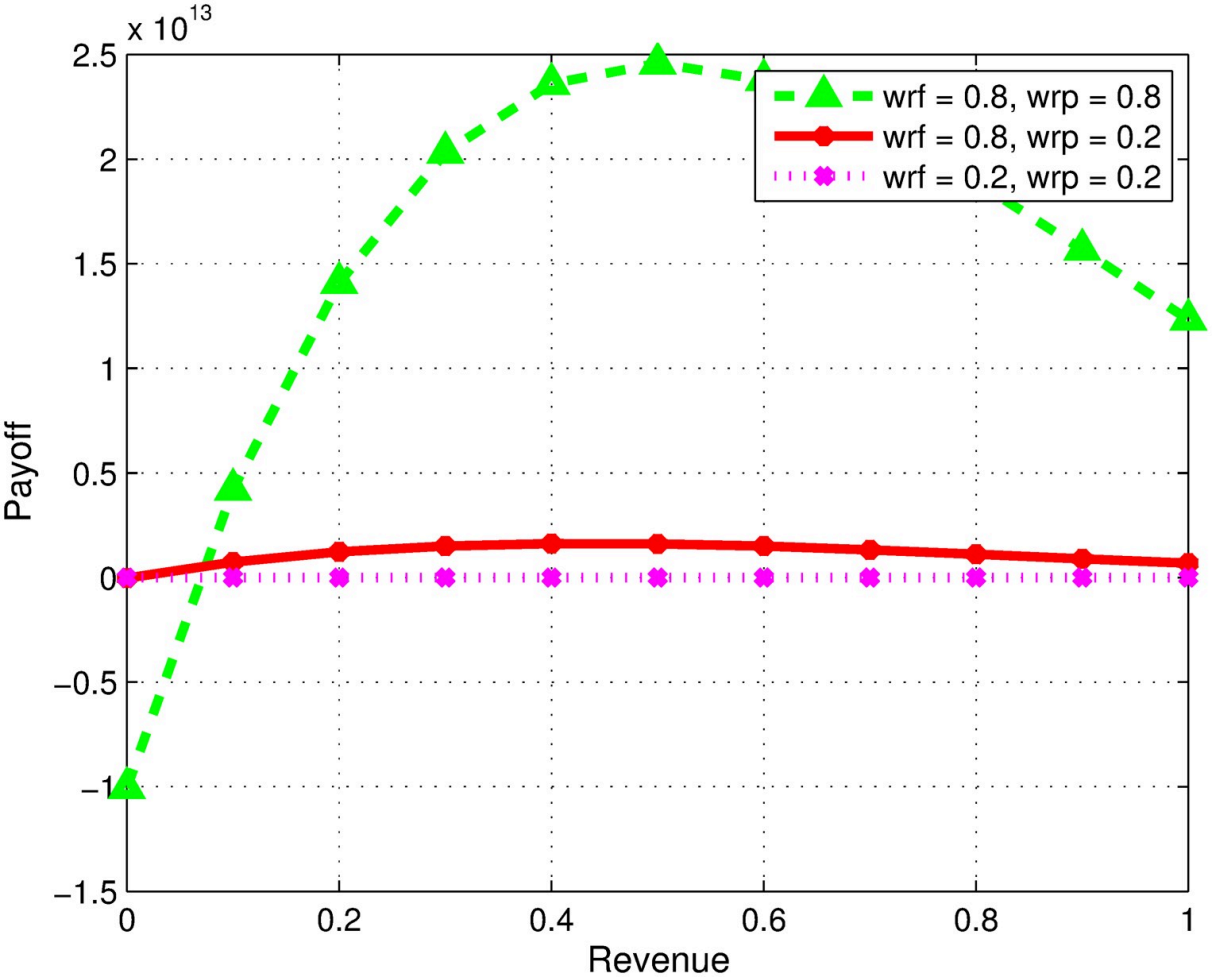
Impact of varying trust.

Preferences of a firm is an important parameter for collaboration formation. Model presented in [13] doesn’t consider the importance of goals of firms towards alliance and the complementarity parameter shows the amount of difference between firms but doesn’t reflect the technical level of firms. As firm with less technical knowledge level may learn more but may not add sufficient value to the product compared to the firm with high technical knowledge level. With change in goal preferences by the firm revenue ratio vary accordingly. Comparing our Fig 1 and 4a with scenario presented in Fig 6. Our model reflects similar behavior if the preferences of both firms are same, but with change in preference parameter and firms having high trust, firm preferring learning should get less revenue of the final profit compare to firm preferring revenue more. Furthermore, the model in [13], does consider the knowledge complementarity *β* as the amount of difference between the knowledge of the two firms. However, it provides no details about the knowledge or the technical level of a single firm.) For instance, one may get the same value of knowledge complementarity when the knowledge level of both the firms is very high and almost similar. On the other hand, the same value might be obtained, if the knowledge level of both the firms is very small and differ by the same value. In reality, a firm with a high technical capability may add more value and may deserve a greater revenue share compare to the other collaborating firm with low technical knowledge.

If the preference of firms are similar that is both firms prefer revenue or learning equally then our model behaviour is almost similar to the model proposed in [13]. However, the model reflect opposing behaviour when one firms prefers revenue and the other firm prefers learning. Therefore, incorporating firms preferences in our work enable us to model more realistic real world situations. Mutual agenda towards collaboration is not always the case in real situations, as firms may prefer different things while collaborating with others.

## Conclusion

Forming an alliance with suitable partner is very important for maximization of collaborative profit. Alliance formation and revenue sharing decision becomes difficult due to involvement of multiple attributes. Previous studies ignore the affect of objectives of firms towards alliance formation. Alliances can be successful only when their formation is mutually beneficial for both the partner firms and are aligned with their personal objectives. In this paper, we modelled the interest of firm towards collaboration by taking revenue and learning as goals for collaboration formation between two firms. The proposed model considers varying parameters along with the same and conflicting goals of firms towards alliance to decide revenue sharing among firms. The proposed model uses Nash bargaining solution to provide optimal solution based on firms’ technical level, cost sharing, cooperation level and knowledge investment and their interest towards collaboration. Analysis of model shows that trust is an important aspect of collaboration as high trust increase learning of firm which can be than used in future project. It also reduces the development cost and added knowledge helps in improving product’s quality. Therefore, it is important to increase the trust level among firms. In practice, the trust among firms improves with the passage of time i.e developing product collaboratively for a long period of time on different projects, or, increase in visits by firms may also help in increasing trust between them. Moreover, knowledge investment and firms’ preferences are also an important aspect of collaboration. The analysis also concludes that a firm interested in learning may agree to collaborate with a lower revenue share depending upon the skills of other collaborating firm.

At the moment, the proposed model is limited to two goals i.e. learning and financial revenue and considers only two firms. This model can be further extended to enable a firm to consider more objectives such as innovation. Furthermore, this work can be extended for alliance that involve two or more firms based on different parameters or contribution of firms to the alliance. As in practice its not always the case that a firm may collaborate with only one firm for developing product. Expertise may be required that are available with separate firms therefore selecting multiple firms in collaboration is also of major concern because each firm may have different perspective’s towards collaboration. Therefore, this work is a solid foundation for further research on multiple objectives where more then two firms are involved.
